# SIMEDIS: a Discrete-Event Simulation Model for Testing Responses to Mass Casualty Incidents

**DOI:** 10.1007/s10916-016-0633-z

**Published:** 2016-10-18

**Authors:** Michel Debacker, Filip Van Utterbeeck, Christophe Ullrich, Erwin Dhondt, Ives Hubloue

**Affiliations:** 1 Research Group on Emergency and Disaster Medicine, Vrije Universiteit Brussel, Brussels, Belgium; 2 Department of Mathematics, Royal Military Academy, Brussels, Belgium; 3 COMOPSMED/B Spec Sp, Medical Component, Belgian Armed Forces, Brussels, Belgium

**Keywords:** Disaster medical response, Pre-hospital disaster management, Victim modelling, Discrete-event simulation, Disaster research

## Abstract

It is recognized that the study of the disaster medical response (DMR) is a relatively new field. To date, there is no evidence-based literature that clearly defines the best medical response principles, concepts, structures and processes in a disaster setting. Much of what is known about the DMR results from descriptive studies and expert opinion. No experimental studies regarding the effects of DMR interventions on the health outcomes of disaster survivors have been carried out. Traditional analytic methods cannot fully capture the flow of disaster victims through a complex disaster medical response system (DMRS). Computer modelling and simulation enable to study and test operational assumptions in a virtual but controlled experimental environment. The SIMEDIS (Simulation for the assessment and optimization of medical disaster management) simulation model consists of 3 interacting components: the victim creation model, the victim monitoring model where the health state of each victim is monitored and adapted to the evolving clinical conditions of the victims, and the medical response model, where the victims interact with the environment and the resources at the disposal of the healthcare responders. Since the main aim of the DMR is to minimize as much as possible the mortality and morbidity of the survivors, we designed a victim-centred model in which the casualties pass through the different components and processes of a DMRS. The specificity of the SIMEDIS simulation model is the fact that the victim entities evolve in parallel through both the victim monitoring model and the medical response model. The interaction between both models is ensured through a time or medical intervention trigger. At each service point, a triage is performed together with a decision on the disposition of the victims regarding treatment and/or evacuation based on a priority code assigned to the victim and on the availability of resources at the service point. The aim of the case study is to implement the SIMEDIS model to the DMRS of an international airport and to test the medical response plan to an airplane crash simulation at the airport. In order to identify good response options, the model then was used to study the effect of a number of interventional factors on the performance of the DMRS. Our study reflects the potential of SIMEDIS to model complex systems, to test different aspects of DMR, and to be used as a tool in experimental research that might make a substantial contribution to provide the evidence base for the effectiveness and efficiency of disaster medical management.

**Electronic supplementary material:**

The online version of this article (doi:10.1007/s10916-016-0633-z) contains supplementary material, which is available to authorized users.

## Introduction

The health community defines a disaster or mass casualty incident (MCI) as an event in which the medical needs exceed, at least temporarily, the response capacities in the affected area, mainly due to a large number of victims and/or severity of the injuries. This imbalance can be due to a quantitative and/or a qualitative shortage of resources (manpower and materials), but also to organizational or operational shortcomings. In this paper, the word “disaster” is used as a synonym for “mass casualty incident or event” [1]. We can no longer rely on our goodwill and good intentions to manage mass casualties in a disaster situation. A different medical approach is needed to achieve the objectives of the disaster medical response (DMR) because of the immediate effects of the disaster on the community and especially on the health care system: the number and variety of injured or ill victims, an initial phase of disorder, the temporary lack of resources and limited output of medical teams directly after the disaster, the necessity to operate in multidisciplinary and complementary teams, and the multiplicity of tasks [2]. The disaster medical response system (DMRS) is an essential part of the overall disaster management system. It is responsible for providing appropriate interventions for the physical, mental and public health of the affected population. The ultimate goal of the DMRS is to minimize as much as possible the loss of life and the suffering of the affected population by managing the temporary imbalance between the immediate health needs and the actual medical response capacity. Successful medical response to a MCI depends on effective and efficient setting of organizational and medical priorities in order to relocate and optimize the utilization of the available resources, and mobilize additional assets [3].

It is recognized that the study of the DMR is a relatively new field. To date, there is no evidence-based literature that clearly defines the best medical response principles, concepts, structure and processes in a disaster setting [4, 5]. Much of what is known about DMR results from descriptive studies and expert opinion [6, 7]. Moreover, databases available for DMR research are underdeveloped, incomplete and inaccurate [8, 9]. Although efforts have been made to collect evidence by systematic reviews on a limited number of medical treatment interventions after the Asian tsunami in December 2004, no such studies have been carried out regarding the effectiveness of the disaster medical system responses on the health outcomes of disaster survivors [10–12]. A prerequisite to adopting any evidence-based approach to DMR is the need to assemble a body of evidence based on the interpretation of empirical data derived from formal research or systematic investigations [5, 9]. Randomized controlled trials (RCTs) are the most robust way to evaluate the effectiveness of medical and operational interventions in response to a MCI. However, it has been considered impossible or ethically inappropriate, or both, to identify experimental and control groups for hypothesis testing in disaster situations [2, 13]. The collection of valid data will always be difficult in disasters, as most healthcare providers prioritize the provision of care to a large number of victims over the documentation of medical and operational decisions [14, 15]. Without a standardized framework for describing and reporting the set of data elements characterizing the medical response and their indicators, it is very difficult to evaluate the impact of response interventions, to compare results of DMR evaluations in different types of disasters, and to perform comparisons across different DMRSs [1, 16].

Just as evidence-based decision-making has gained momentum in medical research, simulation has emerged over the last decades as a useful tool in the study of disaster response. The characterization of the key elements of DMR will facilitate the development of conceptual models which describe a comprehensive approach to manage the medical response to disasters [2]. Traditional analytic methods cannot fully capture the flow of disaster victims through a complex DMRS [13, 17]. Computer modelling and simulation enable to study and test operational assumptions in a virtual but controlled experimental environment. Simulation allows the integration of stochastic and dynamic aspects inherent to the DMR without establishing unrealistic assumptions, offers a large degree of control for the researcher, and enables the study of relationships among any or all variables put into the scenario [13, 18, 19 ]. Computer simulation can provide evidence-based data for an optimal use of resources when applying specific response interventions or procedures, taking into account the contextual factors of the affected area and the specific disaster scenario [2, 20]. Modelling and simulation, if used correctly, in conjunction with available empirical data gathered from lessons learned, can help provide the evidence base for effective and efficient medical response decisions and interventions [17, 20]. In contrast to disaster drills and exercises, simulation can study all possible response situations and test contingency plans in a risk-free environment for both victims and responders [21].

Simulation-based research represented a minimal fraction (4 %) of original research submissions to a dedicated disaster medicine journal in the period June 2013 to May 2014, compared to survey-based research (64 %) and descriptive case series (23 %) [7]. Although modelling and simulation have been used in a variety of applications in the health sector, recent surveys devoted to disaster response showed that DMR research has received little attention from the operations research community until recently [22–24]. Since many recent papers include literature reviews with respect to modelling and simulation of the different issues of the DMR which relate to this study, we will limit the review to enumerating the recent references concerning each of these DMR aspects: victim modelling [17, 25–28], DMR models [17, 28, 29], EMS response [30–32], triage of casualties [27, 33–35], transportation to healthcare facilities (HCFs) [28, 32, 36–38], distribution of victims among HCFs [27, 39–41], medical logistics [42–44], and HCF modelling [28, 45, 46].

A victim-centred model is proposed including the following capabilities: 1) monitoring the health state of MCI victims over time triggered by the elapsed time and/or medical interventions; 2) taking into account the skill level of the responding healthcare responders; 3) taking into account not only the treatment delivery time, but also the treatment effect time; 4) managing the decisions of allocating tasks to available healthcare responders regarding triage, treatment and transportation of injured or ill victims; and 5) addressing the decisions of distributing victims to healthcare facilities taking into account the supervision of patients during the transport and the treatment capacity and capability of the healthcare facilities. This study will be limited to the pre-hospital phase of the acute management of physically injured or ill victims, ie from the scene of the MCI to the admission in healthcare facilities for definitive care. As far as we know, a simulation model with such a level of detail has not yet been proposed in discrete-event simulation (DES) of a MCI response. We hypothesize that such a model can contribute to an important increase of the effectiveness and efficiency of managing victims in the DMR.

## Research Design and Methods

The research objectives of the study were to create a DMR simulation model to be used to test and optimize existing and future medical disaster plans, develop a victim model, a victim creation model and a victim monitoring model, produce a pre-hospital medical response model for disaster situations, and to study the effect of a number of interventional factors on the performance of a DMRS in an aircraft crash simulation at an international airport expressed as the total number of dead survivors. The methodology for the design and development of the structure and processes of the SIMEDIS model is based on data obtained from analysis of responses to past MCIs, quasi-realistic non-computer based simulation exercises, personal experiences of health professionals managing the response to MCIs, and general and specific disaster plans and monodisciplinary intervention plans.

### Conceptual Model

**Fig. 1:**
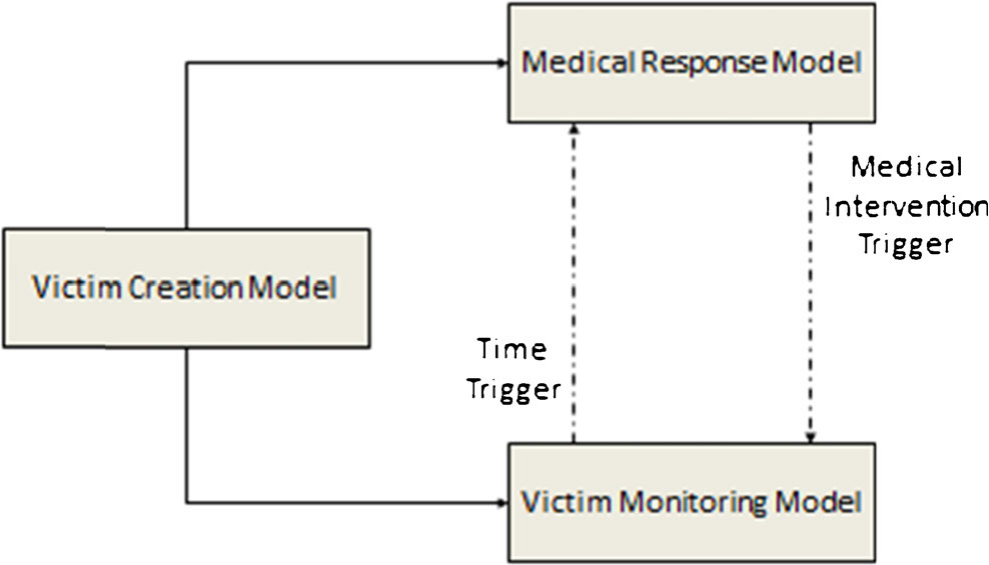
The SIMEDIS model

The conceptual SIMEDIS simulation model is presented in Fig. 1. It consists of 3 interacting components: the victim creation model, the victim monitoring model where the health state of each victim is monitored and adapted to the evolving clinical conditions of the victims, and the medical response model, where the victims interact with the environment and the resources at the disposal of the healthcare responders.

**Fig. 2:**
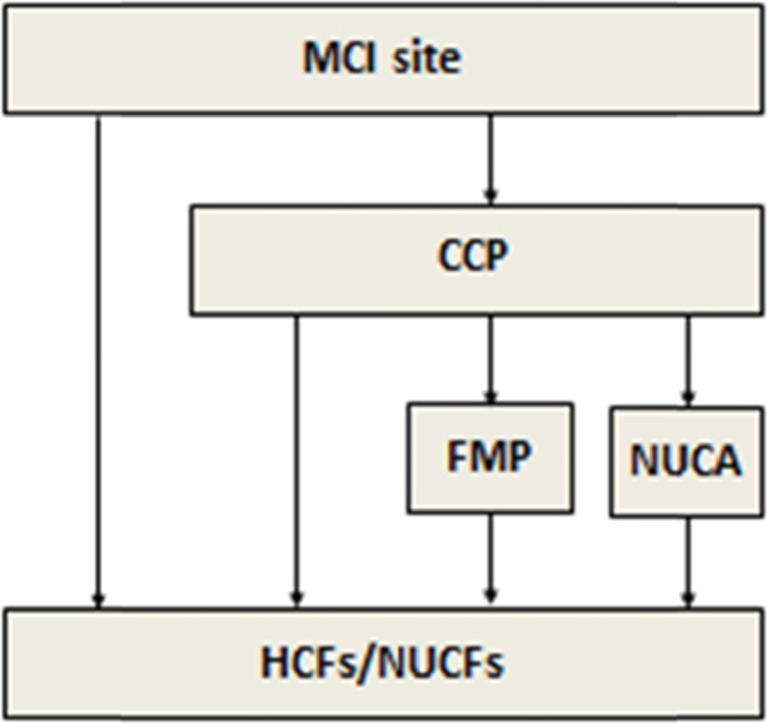
Victim flow in case of an MCI

Since the main aim of the DMR is to minimize as much as possible the mortality and morbidity of the survivors, we designed a victim-centred model in which the casualties pass through the different components and processes of a DMR. The victim flow from the site of an MCI to the definitive HCFs can be described as a series of events. As illustrated in Fig. 2, a victim can be in one of the following environments during the response operation: 1) victims leave the MCI scene without any contact with healthcare responders and self-refer to HCFs or non-urgent care facilities (NUCFs); 2) victims are transferred to a casualty collection point (CCP) just outside the dangerous impact zone by the rescuers; 3) according to the operational policy, victims transferred to a CCP are evacuated to a forward medical post (FMP), a non-urgent care area (NUCA), HCFs or NUCFs; 4) victims transported to an FMP are evacuated to an emergency department of general hospitals or specialist centres (such as trauma centres, burn centres, etc); and 5) victims transported to a NUCA are evacuated to NUCFs such as outpatient clinics, to general practitioners or similar facilities.

**Fig. 3:**
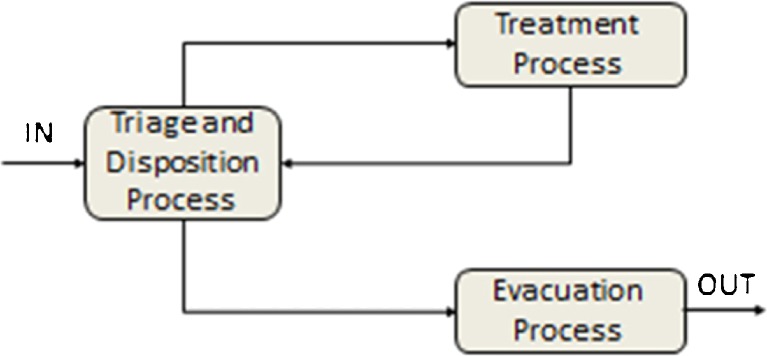
Main processes at each service point of the medical assistance chain

The specificity of the SIMEDIS simulation model is the fact that the victim entities evolve in parallel through both the victim monitoring model and the medical response model. The interaction between both models is ensured through triggers, ie a time trigger and a medical intervention or treatment trigger. At each zone of interest or service point (CCP, FMP, NUCA, HCF or NUCFs), a triage must be performed together with a decision on the disposition of the victims regarding treatment and/or evacuation. For the treatment and evacuation tasks required for a given victim a strict order exists in which they are performed based on a priority code assigned to the victim and on the availability of resources at the zone of interest (Fig. 3). The duration of these tasks are considered to be available upon initialization of the simulation.

### Victim Model

The victim model consists of a description of the victim profile and a set of transitions to move from one clinical state to another. The victim profiles were established using an adapted victim creation template of VictimBase, which was developed by an international consortium of experts in disaster medical management [47, 48]. Each victim profile includes 1) general victim data consisting of all victim parameters that do not change over time or as the result of a medical intervention (eg, identity, gender, anthropometric data, type of injury or illness, body region, diagnosis, medical history, etc); and 2) a set of clinical conditions (CC) or health states of the victim. Each CC consists of a set of parameters. These parameters are arranged into five subgroups: primary survey, secondary survey, diagnostic tests, injury severity scores and triage classifications [49]. The primary survey is composed of the vital parameters such as the respiratory rate, the heart rate, the systolic blood pressure and Glasgow Coma Scale. Several injury severity scores are included in the victim profile. The injury severity score used in this study is defined by the so-called RPM score which consists of respiratory rate, pulse rate and best motor response. The RPM score is the sum of coded values for respiratory rate, pulse rate and best motor response and takes integer values from 0 to 12, with smaller values corresponding to a more severe injury (Table 1) [50].

**Table 1:** RPM score [50]

Coded value	Respiratory rate (per minute)	Pulse rate (per minute)	Best motor response
0	0	0	None
1	1–9	1–40	Extends/flexes from pain
2	36+	41–60	Withdraws from pain
3	25–35	121+	Localizes pain
4	10–24	61–120	Obeys commands

The main triage systems are included in the VictimBase template. The triage categories are specified by subject matter experts (SMEs). The triage levels assigned to the casualties are based on the NATO triage categorization in mass casualty situations [51]. The immediate treatment group (T1) includes those patients with life-threatening injuries and conditions that require immediate life support and urgent hospital admission. These procedures should concern only those patients with high chances of survival. The delayed treatment group (T2) includes hemodynamically stable casualties with injuries and physiologic conditions that will require hospitalization within 2–6 h, otherwise their health state will become unstable. To mitigate the effects of the treatment delay sustaining treatment will be required. The minimal treatment group (T3) is also referred to as the walking wounded. These casualties have relatively minor injuries which require no treatment beyond first aid, and do not require hospitalization. The expectant treatment group (T4) includes casualties who are alive, but with critical injuries and a low likelihood of survival and whose treatment would be time- and resource-consuming. Until the mass casualty situation is under control they will receive appropriate supportive treatment. The evolving clinical conditions are determined by SMEs taking into account, besides the actual CC, time, the medical interventions required, and the availability of health care providers (including their skill levels) and medical equipment or supplies. Each CC of a patient is assigned a survival probability estimate based on the actual RPM score using a logistic regression on data obtained from a retrospective analysis of data from a trauma registry [50]. Sacco’s Delphi estimates of change in survival probability of RPM scores over time were slightly adapted and used to determine the survival time of patients (Table 2) [50].

**Table 2:** Change of survival probability (deterioration rate) of RPM scores in percentage over time, adapted from [50]

RPM	0	1	2	3	4	5	6	7	8	9	10	11	12
0 min	5	9	15	24	35	49	63	75	84	91	94	97	98
30	0	5	5	9	15	24	35	63	84	91	94	97	98
60	0	0	0	5	9	15	24	49	75	84	91	97	98
90	0	0	0	0	5	9	15	35	63	84	91	94	97
120	0	0	0	0	0	5	9	24	49	75	84	94	97
150	0	0	0	0	0	0	5	15	35	63	84	91	94
180	0	0	0	0	0	0	0	9	24	49	75	84	94
210	0	0	0	0	0	0	0	5	15	35	63	84	94
240	0	0	0	0	0	0	0	5	9	24	63	75	94
270	0	0	0	0	0	0	0	5	5	15	49	75	91
300	0	0	0	0	0	0	0	5	5	9	49	63	91
330	0	0	0	0	0	0	0	5	5	5	35	63	84
360	0	0	0	0	0	0	0	5	5	5	35	49	84

**Fig. 4:**
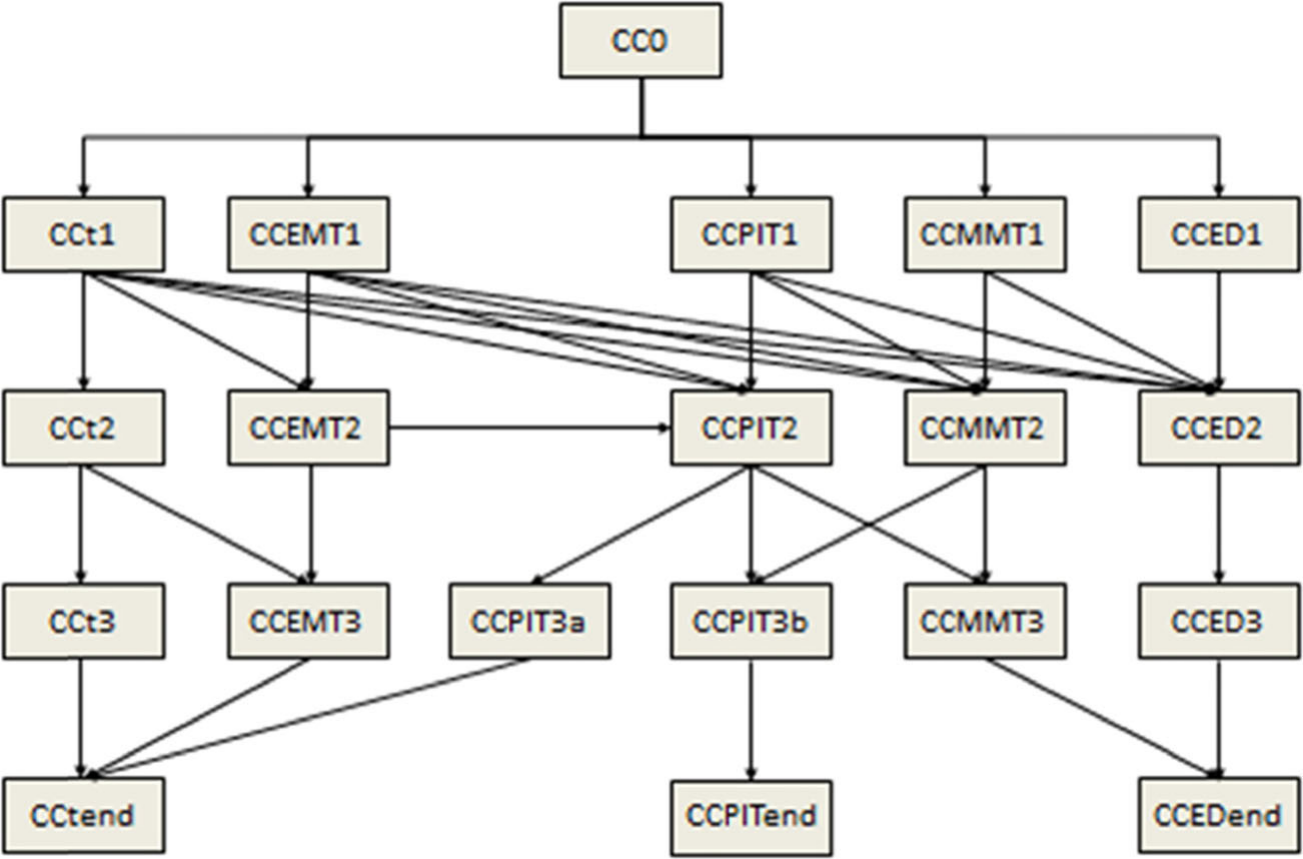
Casualty profile of a victim without any treatment (CCt) or treated by healthcare providers (CCEMT, CCPIT, CCMMT and CCED)

The second element of the victim model is a set of transitions triggered by events that cause the health state of the victim to change from one CC to another. A victim will move to a different clinical state after a certain period of time has elapsed or after a specific treatment has been administered. The application of a medical intervention trigger requires the availability of human and material resources. Each health provider has an associated skill matrix which specifies the interventions he/she is allowed to perform. The skill levels have been defined according to existing national regulations. It is assumed that the mix of healthcare providers has sufficient experience to care for casualties in the field. The list of medical equipment and supplies required for a specific medical treatment is predefined in the model. The initial clinical condition describes the clinical state of the victim immediately after the impact of the hazard. An end clinical condition is a stationary state of the victim, which will not evolve by the passing of time and/or the application of a medical intervention. The transition from one CC to another CC depends on a number of time intervals and the available resources needed to provide the treatment to the victims. The time interval of deterioration of the health state if no treatment is administrated and the time interval of a medical intervention to be effective were determined by medical professionals experienced in prehospital care and disaster medicine. The treatment delivery time interval was based on data published in the medical literature and on experimental studies performed by emergency medicine professionals. Figure 4 shows an example of the different pathways along which a victim can evolve after having received no treatment or a number of medical interventions by different categories of healthcare providers.

The no treatment pathway (CCt) represents the consecutive clinical states the victim will pass through if no treatment is administered. The CCEMT, CCPIT, CCMMT and CCED pathways represent the consecutive health states the victim will pass through if a treatment is administered respectively by emergency medical technicians (EMT), nurses or paramedics (PIT), mobile medical teams (MMT) composed of an emergency physician and nurse, and in the emergency department (ED). The vertical transitions in the different treatment pathways assume that the started treatment will be continued during a certain time interval. The oblique transitions between the different treatment pathways assume that healthcare providers with higher skills become available and initiate a treatment according to their skill level. The oblique transitions between treatment pathways and end clinical conditions assume that either the administered treatment was unable to stabilize the victim leading to his/her death or was able to stabilize the clinical condition of the victim. Administration of care will result in a decrease in morbidity (ie a better injury severity score for example a higher RPM score) and an increase in the amount of “life-time” (ie a higher survival probability). On the contrary, a delay in treatment, either by queuing or lack of staff and/or supply resources, will reduce the amount of “life-time” (ie a lower survival probability) and increase the morbidity (ie a worse injury severity score for example a lower RPM score). The detailed elaboration of the victim profiles allows us to define the exact health condition of the victims over the course of the simulated response.

### Victim Creation Model

**Fig. 5:**
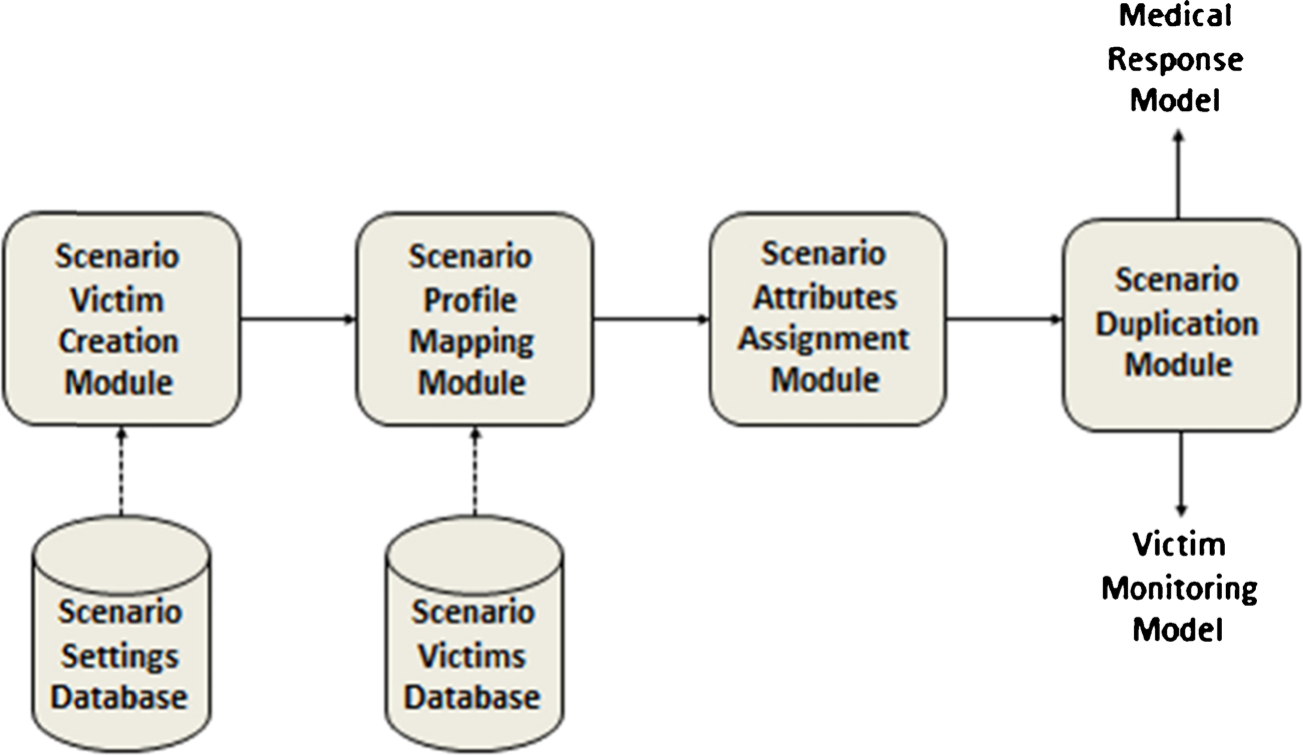
Victim creation model

The victim creation model consists of a scenario victim creation module, a scenario profile mapping module, a scenario attribute assignment module and a scenario duplication module (Fig. 5).

The victim creation module generates the number and types of injured victims included in the scenario. Each victim receives a victim profile according to the nature of his/her injuries in the scenario profile mapping module and a number of other attributes in the scenario attribute assignment module, for example if the victim needs to be treated in a burn or trauma centre. The scenario duplication module creates two copies of the victim entity which will be sent to the victim monitoring model and the medical response model.

### Victim Monitoring Model

**Fig. 6:**
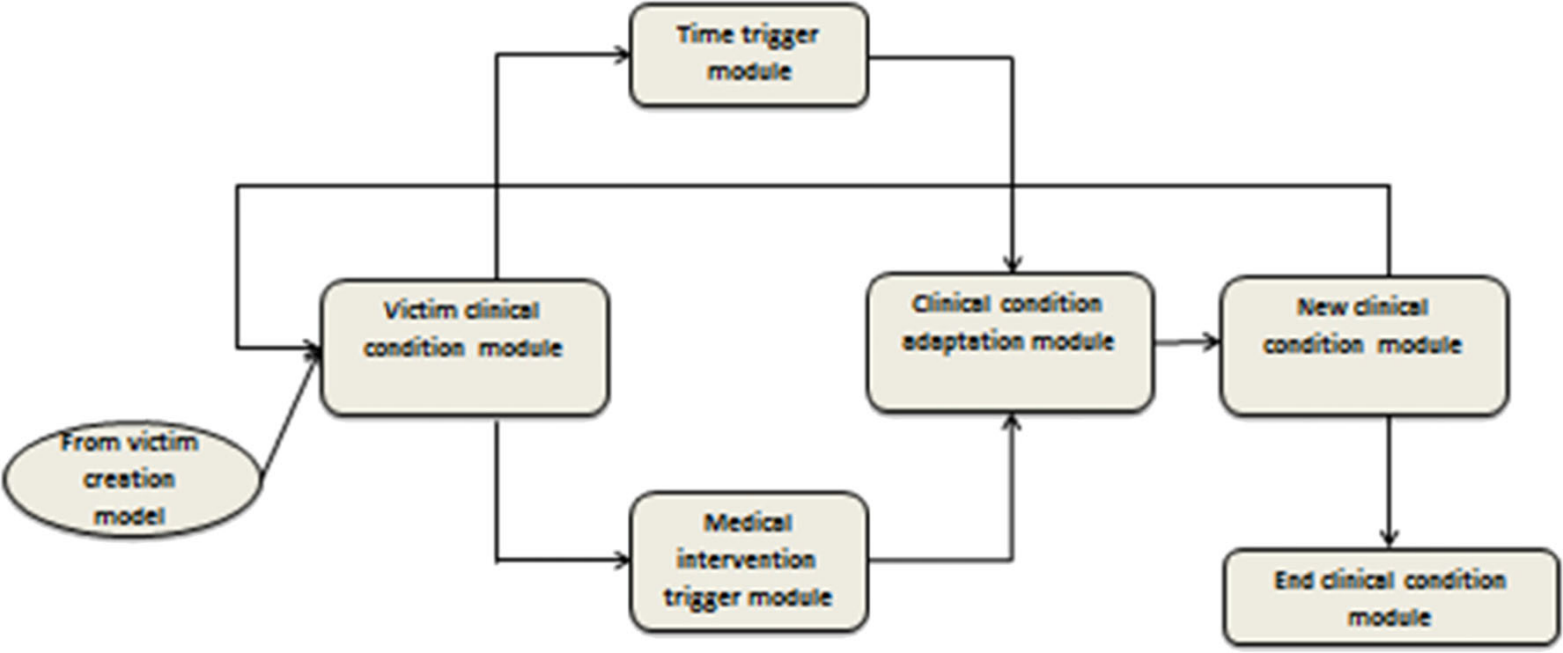
Victim monitoring model

The victim monitoring model manages the dynamic evolution of the health state of each victim in the disaster scenario and the transitions from one CC to another CC. The clinical condition adaptation module adapts continually the health state of the victims via a time or medical intervention trigger (Fig. 6).

### Medical Response Model

The medical response model represents the environment of the responders in order to rescue, triage, stabilize and evacuate victims.

A typical medical response model consists of the environment (geographical areas relevant to the scenario, time), the resources at the disposal of the disaster response system (manpower, equipment, supplies, means of transportation, hospital treatment capacities, etc), a set of medical/operational decision rules and the localization of victims as they are evacuated from one area to another.

**Fig. 7:**
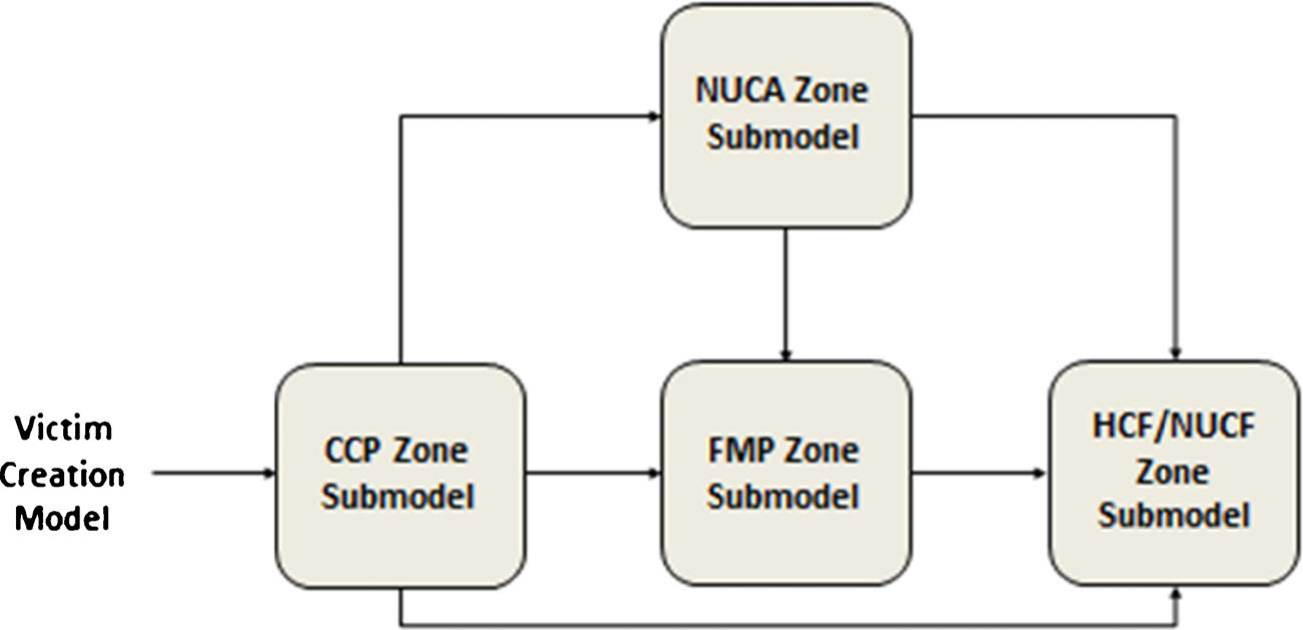
The SIMEDIS medical response model

Four zones of interest or service points are included in the medical response model: the disaster site with the CCP, FMP, NUCA, the ED of the receiving HCFs and the NUCFs (Fig. 7).

**Fig. 8:**
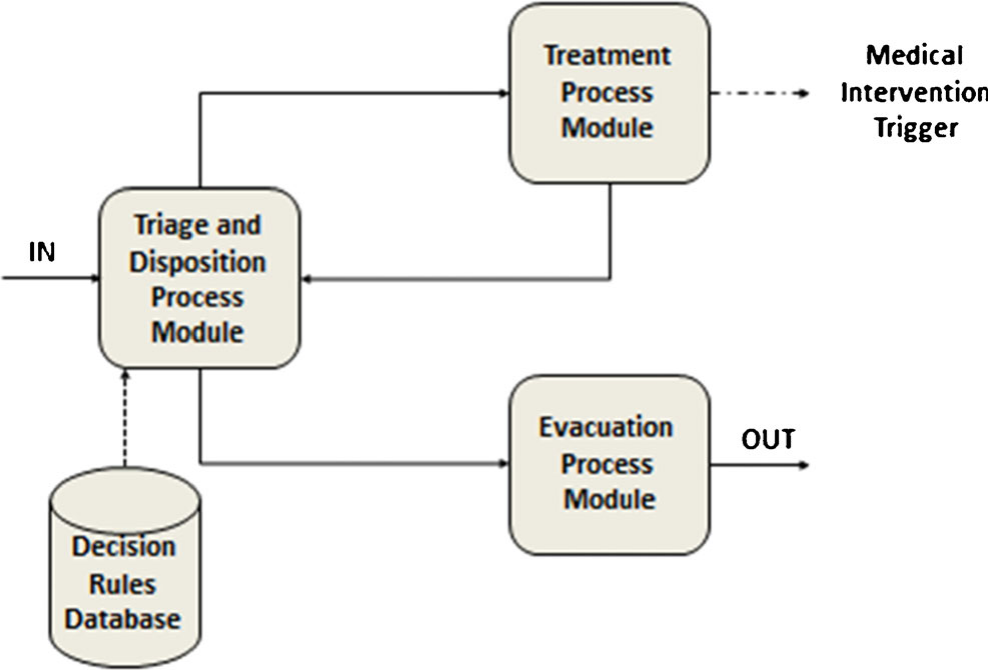
The SIMEDIS medical response model processes

The management of the flow of MCI victims at the different service zones consists of three processes: triage, treatment and evacuation of the victims. The fluent integration of all three processes is essential to ensure an appropriate and efficient management of the patients along the chain of medical assistance (Fig.8).

The detailed description of the translation of the conceptual model into a computer simulation model can be found in Appendix 2. The outcome measure for this model is the overall mortality among the survivors.

### Model Verification and Validation

It has been suggested that various techniques should be applied for verifying and validating the simulation model [13, 52]. The accuracy of the programming and implementation of the computerized simulation model was verified and accomplished by debugging errors. Animation was used to visualize the flow of the MCI victims through the DMRS in order to check that the model functions according to the specifications [48]. Finally, the accuracy of the output was verified in the log reports using detailed timelines to review the various processes in all sub-models and charts of output data were generated in order to indicate possible problems in process logic or resource utilization. From this analysis we concluded that the SIMEDIS model is a correct translation of the conceptual model. An experimental or operational validation is impossible, because the MCI cannot be created in reality due to ethical considerations and because historical data of operational interventions of the medical response do not exist or are not documented with enough detail. Moreover they may not represent the current scenario or do not reflect the actual DMRS under investigation. To validate the correctness of the model’s logic, the movement of patients and vehicles and the occurrence of every event in the model were tracked in the simulation log. The model behaviour was evaluated in a pilot study in which the response to normal as well as extreme situations was examined in order to determine if the model output behaves as expected [53]. Face-validity of the model was tested by users and subject matter experts who assessed the accuracy and consistency of the simulation output and outcome data compared to the real-world system. All these validation methods testify to correctness of the SIMEDIS model in adequately representing the different aspects of a DMR to an MCI with respect to the research requirements.

## Case Study

The aim of the case study is to implement the SIMEDIS model to the DMRS used at Zaventem airport and to test the medical response plan to an airplane crash simulation at the airport. In order to identify good options for responding to this type of MCI, the model then was used to study the effect of the following interventional factors on the performance of the DMRS: operational policy (“scoop-and-run” versus “stay-and-play”), triage procedures, search and rescue (SAR), quantity and quality of resources, victim distribution among HCFs, medical supervision of victims during transport, and HCF treatment capacity. The motivation for the setting stems from the fact that the Medical Component of the Belgian Armed Forces is responsible for the medical command and coordination of the preparedness of and the response to an MCI at Zaventem airport.

### Scenario

The MCI is an airplane crashing on the runway when landing at the airport, but without a fire. The weather conditions are normal. There were 250 occupants in the plane with 205 injured victims (26 T1, 62 T2, 113 T3 and 4 T4 patients), 5 immediate fatalities and 40 uninjured victims. The mix of injury type and severity and the average percentage of patients in each triage category are based on published data of the International Civil Aviation Organization and historical airplane crashes at airports in the medical literature [54–57] The Belgian EMS system consists of three skill levels: Basic Life Support (BLS) ambulances with 2 Emergency Medical Technicians (EMTs), Paramedic Intervention Team (PIT) ambulances with 1 EMT and 1 nurse or paramedic, and Mobile Medical Team (MMT) with 1 emergency physician and nurse. If a patient needs Advanced Life Support (ALS) care during the transportation to a HCF, the MMT and medical equipment from the emergency vehicle are transferred into the BLS ambulance. The disaster medical response processes and structures to be implemented following an airport aircraft crash are extracted from general and specific emergency and intervention plans including the medical intervention plan (MIP) and the disaster plan of Zaventem airport [58–61].

In a disaster situation emergency medical care is first supplied by local and regional EMS and Red Cross rapid intervention teams (RIT) and subsequently, additional support is solicited from neighbouring regions. During an MCI, the day-to-day emergency calls within the region also must be covered. Consequently, only half of the available EMS resources within the region will be dispatched to the incident scene. The pre-hospital operations are initiated upon activation of the MIP and comprise all actions to limit the health impact of the MCI: dispatching resources, rescue, triage, treatment, evacuation and distribution of the victims among appropriate HCFs. When the medical intervention plan is activated, a fixed number of MMTs, ambulances, RITs of the Red Cross are dispatched to the disaster site. In an airport MCI, 4 MMTs, 5 ambulances and 2 RITs will immediately be dispatched to the scene of the incident. These units are assigned to the different service points at the site (Medical Command Post, CCP, FMP, NUCA). Reinforcements are dispatched from the same or neighbouring regions according to the health needs assessment. A number of EMS teams will travel to the incident site, stay and stabilize victims at the scene or travel back and forth between the scene and the HCFs to transport casualties, depending on the decisions of the medical commander on the scene. The first MMT that arrives will assume the role of medical commander on the scene of the incident. He/she is responsible for assessing the health needs of the situation, supervising all medical response activities at the incident site, confirming the number and types of injured and/or ill victims to the dispatch centre and hospitals, establishing the different operational zones or service points such as CCP, FMP and NUCA, and designating the triage, treatment and evacuation officers. The increase of the pre-hospital surge capacity (transportation means, staff, medical equipment and supplies) are implemented by the medical incident commander and the EMS dispatch centre in accordance with the approved protocols included in the MIP. All these activities are included as rules in the simulation model.

The disaster plan of Zaventem airport includes following procedures and structures in case of an aircraft crash. The MMT from the Military Hospital assumes the role of medical commander at the incident scene and does not participate in the medical care or transport of the victims. Search and Rescue (SAR) is performed by fire fighters from the airport fire department with reinforcements from 3 neighbouring departments. A preliminary triage occurs during the SAR process. The slightly injured or ambulatory victims will self-evacuate out of the airplane and are escorted to the CCP. The seriously injured or non-ambulatory survivors will be extricated out of the crashed plane and are transported to the CCP by the fire fighters. The second MMT that arrives at the incident site moves to the CCP in order to start the primary triage on the scene. Once the primary triage is finished the emergency physician and nurse move to the FMP to provide treatment to injured victims or to supervise patients during their transport to HCFs. A physician and nurse of the medical service of the airport also move to the CCP where they will perform primary triage and afterwards will help caring for the slightly injured victims in the NUCA. The CCP is located at 25 m of the crashed plane as there is no risk for possible exposure to fire and smoke. On arrival at the CCP victims are already sorted in an urgent and non-urgent category. The non-urgent group is transferred to the NUCA. The seriously injured are triaged and transported by 5 ambulances to the FMP according to their priority level. The premises of the airport fire department are transformed into an FMP and equipped with medical supplies which are stored in advance in these premises and reinforced by medical logistics supplied by the MMTs and 2 RITs of the Red Cross. The third and fourth MMTs move to the airport fire department and start the setup of the FMP. Treatment of victims can start 8 min after arrival of the MMTs as was demonstrated in several exercises to test the airport disaster plan. All the additional MMTs will report to the FMP and help in the treatment of injured victims at the FMP or in the supervision of seriously injured during the transport to HCFs. Seriously injured patients will be distributed in a rotational way among the HCFs taking into account the treatment capability and capacity of each HCF. Non-urgent injured victims are transported by airport buses from the CCP to the NUCA where they are re-examined by staff of the Red Cross and the Medical Service of the airport. Patients whose health state meanwhile has deteriorated will be transferred to the FMP. The other victims will be cared in the NUCA and sent home or transported to NUCFs in minibuses of the Red Cross for definitive treatment. Since the airport premises are a closed area, patients cannot self-refer to HCFs or NUCFs. The operational assumptions and all specific input data of the experimental design can be found in Appendix 3.

### Experimental Design

In order to identify good options for responding to this type of MCI, the simulation model then was used to study the effect of the following interventional factors on the performance of the DMRS: operational policy, triage procedures, search and rescue, quantity and quality of pre-hospital resources, victim distribution among HCFs, level of supervision of victims during transport, and HCF treatment capacity.

### Search and Rescue

All victims who can walk are considered as uninjured or slightly injured (T3) and have evacuated the plane within 3 min after the crash and are escorted to the CCP. Non-ambulatory patients are removed from the plane and transported to the CCP by firemen in a random order. As a result of the SAR process the survivors are sorted in seriously injured (urgent) and slightly injured (non-urgent) victims, a so-called preliminary triage. The SAR of the non-ambulatory victims starts 5 min after the crash. Two additional holes in the airplane fuselage are made by fire fighters after 21 min. Three SAR rates have been studied as mentioned in Table 1 of Appendix 3.

### Operational Policy

The processes, interactions and relations between the service points are determined by the operational policy: the scoop-and-run or the stay-and-play policy. If the scoop-and-run policy has been selected by the medical commander, all injured victims are directly evacuated from the CCP to HCFs and NUCFs. The care of the urgent victims will start during the transportation to HCFs according to the skill level of the healthcare provider supervising the patient. All MMTs, except the medical commander and the triage team at the CCP, are employed to supervise the seriously injured victims during the transport to HCFs. In the stay-and-play policy, the severely injured victims are transported from the CCP to the FMP to be stabilized before being evacuated to appropriate HCFs. The slightly injured patients are transferred to the NUCA and subsequently sent home or transported to NUCFs.

### Triage Procedures

Triage is a key factor in managing the victim flow in the treatment and evacuation process of a MCI. The triage levels assigned to the casualties are based on the NATO triage categorization in mass casualty situations [51]. If triage is performed urgent casualties will be assigned a priority code which reflects the triage level, the injury severity score (RPM score) and the time of arrival in the queue. For example a T1 victim with an RPM score of 6 and arrival time in the queue of 45 min takes a priority code of 106045. A T2 victim with a RPM score of 11 and arrival time in the queue of 110 min takes a priority code of 211110. The victims with the lowest priority code, has the highest priority with respect to treatment and evacuation. If the urgent victims are not triaged their priority for treatment and evacuation is based on the first-in-first-out principle.

### Quantity and Quality of Pre-Hospital Resources

The majority of transportation MCIs, including airplane crashes, are self-contained. There is thus no real lack of medical resources in the community or region, but rather a problem of their effective mobilization and deployment in order to respond to an immediate short term surge through maximizing and reallocating existing pre-hospital resources [62, 63]. Moreover, the region’s medical surge capacity is normally determined at the time of the planning. Consequently, a deterministic approach is used for modelling the various resources required for the DMR. We used the actual available resources in the community and regions, including EMS staff, medical equipment and supplies and transportation means. The initial dispatching of EMS units to the disaster site is made according to the MIP, ie 5 BLS ambulances and 4 MMTs of which one will function as the incident medical commander. We assume that locally and regionally there are 38 ambulances and 10 MMTs available to respond to the incident. The ambulances are initially dispatched from one of the 32 EMS stations which are distributed over the local area and the surrounding regions. The rest of the pre-hospital resources is assigned to the day-to-day emergency calls. Table 2 of Appendix 3 shows the different levels of resources studied in the scenario. Treatment and transportation of victims cannot be provided unless human and material resources are available. It is assumed that all care providers have experience with caring for casualties in the field. We assume that only seriously injured victims are admitted to HCFs for definitive treatment and consequently ambulances will only be used to transport severely injured patients. Each ambulance can carry 1 T1 or T2 patient at a time and each patient is directly transported to an appropriate HCF after having been picked up at the CCP or FMP according the operational policy. The loading of a patient in the FMP can only start after expiration of the treatment delivery time. Once the pickup decision is made, no change is allowed even if a patient with a higher evacuation priority becomes available in the evacuation queue. If emergency physicians and/or nurses are in an idle state at the FMP, they will be used in supervising T1 or T2 patients during their transport to HCFs. Ambulances will travel along a calculated shortest path to the disaster site and from the CCP or FMP to HCFs. A target HCF will be chosen following the distribution policies of the simulation scenario under study. The operational time intervals are determined empirically by using historical statistical data of the regional EMS department or data collected during the yearly exercises to test the airport disaster plan. The response and transportation time interval (expressed in min) are calculated by multiplying the distance (km) between pairs of pre-determined points with the average speed (km/h) of the ambulances and MMT vehicles and subsequently divided by 60. It should be noted that the average speed data used referred to EMS units travelling to respond to everyday emergencies. We assume that a responder unit will always take the shortest path through the availability of navigation routing technology. The different time intervals are included in Table 3 of Appendix 3. When using the stay-and-play policy, the travel time interval from the CCP to the FMP is 1 min in this specific scenario.

### Victim Distribution among HCFs

Two victim distribution policies are studied in the experimental design. Patients are allocated to the nearest HCF first until its surge capacity is reached and are then sent to the next closest HCF and so on, or victims are distributed in an alternating way among the different HCFs, the so-called leap-frogging approach or round-robin fashion based on their treatment capacity.

### Level of Medical Supervision during Ambulance Transport

Four different levels of medical supervision have been studied with respect to the medical supervision of the injured casualties during transport of victims, using EMTs, emergency nurses or emergency physicians. Table 4 of Appendix 3 shows the different combinations used in the study.

### HCF Treatment Capacity

In order to control the victim flow, it is essential to know the treatment capacity and capability of the HCFs, in particular just after the onset of the MCI when HCFs have not yet activated their disaster plans. The number of patients a HCF can treat at any given time has been determined in the regional MIP (Table 5 of Appendix 3). Once this number of casualties has been transported to an HCF, the treatment capacity is defined as a number of patients per hour as indicated in the disaster plan of the HCF (Table 6 of Appendix 3).

## Results

We have conducted an extensive empirical evaluation of the impact of various interventional factors in the DMR on the mortality of the survivors of an airplane crash at an international airport. The impact of seven DMR interventional factors on the total number of deaths was investigated using a full-factorial experimental design, leading to 1152 different design settings. Thirty replications of each simulation scenario have been carried out for a total of 34,560 data points. The data were analyzed using descriptive statistical methods and univariate analysis of variance for the main effects. Post-hoc Scheffé tests were used to determine which variables caused differences in the one-way ANOVA. Table 3 summarizes the descriptive statistics obtained considering every input variable separately.

**Table 3:** Descriptive statistics of every input variable separately. (HCF: healthcare facility, TC: treatment capacity, SAR: search and rescue)

Statistics for Total	All cases	Policy		Triage		Supervision Transportation				Distribution HCF	
Number of Dead	n/a	S&P	S&R	False	True	Low	Medium	Normal	High	False	True
Average	19.90	22.15	17.66	22.64	17.16	21.60	18.85	18.82	20.35	19.50	20.31
Median	19.00	23.00	18.00	24.00	18.00	23.00	19.00	19.00	19.00	19.00	20.00
Std Dev	4.71	3.75	4.50	3.58	4.07	4.24	5.20	5.23	3.32	4.89	4.49
Min	5.00	15.00	5.00	13.00	5.00	10.00	5.00	5.00	15.00	5.00	7.00
Max	29.00	28.00	29.00	29.00	28.00	29.00	28.00	28.00	28.00	28.00	29.00
Statistics for Total	All cases	Pre-hospital Resources				HCF Treatment Capacity			SAR		
Number of Dead	n/a	Low	Medium	Normal	High	Low	Medium	High	Low	Medium	High
Average	19.90	19.97	19.79	19.97	19.89	19.90	19.90	19.90	21.83	19.19	18.69
Median	19.00	19.00	19.00	19.00	19.00	19.00	19.00	19.00	22.00	19.00	18.00
Std Dev	4.71	4.71	4.71	4.64	4.78	4.71	4.71	4.71	3.27	4.96	5.06
Min	5.00	6.00	6.00	5.00	5.00	5.00	5.00	5.00	13.00	5.00	5.00
Max	29.00	29.00	28.00	29.00	28.00	29.00	29.00	29.00	29.00	29.00	29.00

### Operational Policy

There is a significant difference in average mortality (*p* < 0.001) between the two operational policies in this specific scenario, as determined by one-way ANOVA: 17.66 deaths with the scoop-and-run policy versus 22.15 in the stay-and-play policy. However, looking at the frequency distribution of the subtraction of the number of deaths in de stay-and-play policy and the number of deaths in the scoop-and-run policy [Δdeath = Deaths (Policy = S&P) – Deaths (Policy = S&R)], we found that in 11 % of the 1152 scenarios, the stay-and-play policy is the best option to minimize the number of deaths. Moreover, this result may not be generalized to other MCIs or DMRS, since for instance in the pilot study to validate the model the stay-and play policy had a lower mortality in a pileup scenario [53].

### Triage Procedure

There is a significant difference (*p* < 0.001) in the total number of deaths between performing and not performing triage as determined by one-way ANOVA: 17.16 deaths with triage and 22.64 deaths without triage. To the best of our knowledge, this study shows for the first time that triage can decrease the mortality in a specific MCI scenario, taking into account a number of DMR interventional factors. The in-depth analysis of the impact of the interactions of triage with the other interventional factors will be the subject of further research.

### Search and Rescue

The average mortality between the 3 rates of SAR or in other words between the 3 victim flows to the CCP, is significantly different (*p* < 0.001), as determined by one-way ANOVA: 18.69 deaths for a high flow of victims, 19.19 deaths for a medium flow of victims and 21.83 deaths for a low flow of victims. A post-hoc Scheffé test confirmed the difference in mortality between the 3 groups.

### Quantity and Quality of Pre-Hospital Resources

Although the mean number of deaths is almost equal between the 4 levels of pre-hospital resources, the one-way ANOVA test shows a significant difference (*p* < 0.001) between the 4 groups. However, a post-hoc Scheffé test indicates that this is only the case between the low and medium group and the medium and normal group. The reason is that more MMTs (7 instead of 3) are dispatched in the first wave (3–15 min after the crash) to the MCI site, allowing an earlier stabilization of a larger number of seriously injured victims in the FMP or during the evacuation to HCFs.

### Medical Supervision during Ambulance Transportation

There is a significant difference (*p* < 0.001) in average mortality between the 4 levels of supervision as determined by one-way ANOVA. The post-hoc Scheffé test confirms the significant difference between the groups except for the comparison between the low and normal supervision groups (*p* = 0.837). Supervision by EMTs only has a negative impact on the total number of deaths when the scoop-and-run policy is applied, as no advanced trauma life support can be started in seriously injured patients during their ambulance transportation. The higher mortality in the group of survivors whose medical supervision is performed by a team of an emergency physician and nurse is due to the fact that critically injured patients die while they are waiting for such a team to become available.

### Distribution of Victims among HCFs

The rapid and efficient transport of seriously injured victims to the appropriate level of care has been identified as a key factor in the successful response to a MCI [64]. Although it has been recommended to distribute disaster victims among the different HCFs available in order to not disproportionately overload one HCF, historical data show that very often the majority of victims were transported to the closest hospital(s) [6]. The distribution of the victims to the HCFs nearest to the disaster site has a significantly lower average mortality (*p* < 0.001) as determined by one-way ANOVA than the allocation of victims in an alternating manner among the different HCFs of the region. This is due to the fact that the total surge capacity of these closest HCFs was adequate to treat the number of seriously injured victims in this specific scenario.

### HCF Treatment Capacity

The HCF treatment capacity has no impact on the average mortality of the survivors in this specific MCI scenario, due to the fact that airplane crashes are self-contained MCIs and do not cause a real lack of resources in HCFs of the community or region, once the disaster plans of the HCFs have been activated.

The percentage of explained variance (r squared) of this analysis of variance model is 71.9 %. Although the effect of all the interventional factors on the mortality of the MCI victims, except the HCF treatment capacity, is highly significant, their contribution to the impact on the mortality shows large differences. Triage contributes 33.8 %, operational policy 22.7 %, SAR 8.5 % and supervision during transport 6 % to the total number of deaths. The other 2 factors contribute less than 1 % to the mortality. In order to obtain the optimal medical response in this specific scenario of an airplane crash at an airport, the medical commander should use a scoop-and-run policy, order to perform triage, request from the rescuers a high SAR rate, and provide a medical supervision of the seriously injured victims during transportation carried out by a paramedic, emergency nurse or physician.

## Discussion

Disaster planning plays an important role in the management of the health impacts in MCIs and particularly in balancing medical resources availability and demand in an efficient manner [6, 65]. The DMR planning is only as good as the assumptions on which it is based. Many of these assumptions are incorrect and/or not based on systematically collected evidence [6, 66]. Evidence-based research that analyses the effectiveness and efficiency of a DMR on the health outcomes of disaster survivors is rather scarce [10–12]. One of the reasons is the fact that RCTs have been described as unable to accommodate the complexity that characterizes DMR and are impossible or ethically inappropriate, or both, to be carried out in disaster situations [2, 13]. Nevertheless, there is an increasing awareness among the health and medicine community for the need of research that supports valid, reproducible conclusions on the effectiveness and efficiency of the DMR [2, 5, 67].

Traditional analytic methods cannot easily capture the flow of disaster victims through a complex DMRS [13, 17]. Therefore, we designed and tested a DES tool for assessing the management of injured survivors of an sudden onset MCI in the pre-hospital part of the medical assistance chain and used an airplane crash at an international airport as a case study. Computer modelling and simulation enable to study and test operational processes in a virtual but controlled experimental environment [2, 20]. The identification and use of relevant indicators is a crucial part in determining the impact of medical and operational interventions in disaster response [16]. The literature generally distinguishes between process or performance indicators and impact or outcome indicators. Performance indicators concern both the output of the interventions conducted and the process of implementation of the interventions. Outcome indicators measure the actual achievement intended by the interventions in the disaster response [2]. Disaster medical services have a tendency to measure their interventions in terms of process outputs (e.g. how many ambulances were dispatched to the disaster site) rather than assessing the health impact these interventions have on the disaster victims they are assisting. The DMR management must be assessed from the point of view of the impact of the interventions on the total number of casualties with the end goal of minimizing the human cost of disasters rather than from the point of view of the DMRS’s needs [1]. Process evaluations within simulations explore the implementation of interventions and help the interpretation of the outcome results. Process evaluation can help to distinguish between interventions that are badly conceived and those that are poorly performed. Nevertheless, by integrating process and outcome evaluation within simulations, we can maximize the ability to interpret the experimental results according to empirical evidence. The SIMEDIS simulator is capable of assessing the performance of a DMRS by using both process and outcome indicators and can therefore be used as a tool for research purposes in order to provide the evidence base for effective and efficient medical response decisions and interventions. This paper does not attempt to develop a tool that can be used in a decision support system in real time. Our goal is to develop a relatively simple simulation model that captures the dynamics of the essential components of the victim flow in a DMRS in order to test interventions included in existing disaster plans as well as alternative assumptions in order to propose more efficient solutions taking into account the type of MCI and the context of the affected environment.

The SIMEDIS simulator consists of three interacting components: the victim creation model, the victim monitoring model and the medical response model. Since the main aim of DMR is to minimize as much as possible the mortality and morbidity of the survivors, we designed a victim-centred simulation model in which the casualties drive the simulation through triggering the various medical processes at defined service points within the medical assistance chain. SIMEDIS is unique in several respects. The victim creation model generates very detailed victim profiles and the victim monitoring model continually updates the clinical conditions of victims during a simulation, triggered by the elapsed time if no treatment is provided or by the medical interventions administered by healthcare responders. Various methods have been used to determine how the health state of the casualties evolves over time such as the Sacco’s triage method [STM] model [27, 39, 50], the Markov chain methodology [28, 68, 69], health state measures [26, 70], time dependent mortality curves [71], and data fusion [72]. The SIMEDIS model uses a deterministic approach to determine the evolving CCs of the casualties, whereby for each CC all clinical parameters, including the derived RPM scores, are pre-determined by SMEs. We used the STM deterioration model for calculating the survival time of a seriously injured survivor. This approach also allows us to model the worsening of the CC over time if no treatment is provided or a possible improvement of the CC after administration of a medical treatment taking into account the skill level of the healthcare provider. As far as we know, such a level of detail has not yet been proposed in a DES of a medical response to an MCI. This method is time-consuming, as it requires the modelling of the complete health history of the victim and the determining of the different transitions from one CC to another, including the time interval between the different CCs. The benefit of this approach is that the health state is always based on the current clinical parameters and not on prediction probabilities. Moreover, these victim profiles can be stored in a victim database and re-used in other MCI scenarios with similar injury types and severities.

As illustrated in Fig. 7, the environment or service points in the medical response model represent the spaces through which the victims flow in the medical assistance chain: at the disaster site (CCP), FMP, NUCA, HCF or NUCF, or in ambulances. The processes included in the medical response model represent the interventions which are required to manage the casualties in the field: triage, disposition, treatment, distribution and evacuation of the injured survivors. The SIMEDIS simulator provides the victims with a priority code based on the triage category, the RPM score and the waiting time in the queue. The victim will trigger, according this priority code, a disposition decision with respect to the treatment and evacuation at each service point taking into account the availability of the pre-hospital resources and the HCFs’ treatment capacity and capability. The resources represent the assets (people, equipment, supplies, transportation means) required to accomplish the processes. The simulation terminates when the casualty is admitted in the ED of an HCF. The subsequent services, such as ED and inpatient care are not within the scope of this paper, but the same processes could be applied at the in-hospital service points.

The case study has shown that the SIMEDIS simulator allows for testing the impact of several interventional factors on the DMR in a specific MCI scenario and can provide useful insights into the factors which have an important effect on the performance of the DMRS allowing planners to establish more robust plans to deal with these types of MCIs. The main reason why we used pre-determined deterministic and not stochastically generated values for input variables and data is that the variance of the simulation results is drastically reduced.

The SIMEDIS model can contribute to further experimental research on the effectiveness and efficiency of a DMR and can provide evidence-based data for an optimal use of resources when applying specific response interventions in order to define best practice taking into account the contextual factors of the affected area and the specific disaster scenario.

Areas of future work that the authors are currently pursuing include the development of methods that build stochasticity directly into the SIMEDIS model, the extension of the model to the emergency department and in-patient services of HCFs, the application of the simulation model to a variety of potential disaster scenarios, and the analysis of various DMR management alternatives.

## Limitations

There is currently no evidence available demonstrating that the STM survival probabilities and deterioration rates of patients from day-to-day emergencies can be extrapolated to disaster survivors or that the CCs of disaster victims evolve in the same manner as everyday trauma patients.

The SIMEDIS simulator uses empirical data of a specific DMRS and environment. Moreover, disaster plans must be updated regularly because of changes in the DMRS and/or environment. Thus, some input parameters of the simulation model should be updated on a regular basis and simulations should be repeated at regular intervals to account for these changes. It is our intention to develop a stochastic version in the near future.

The inability to operationally validate the SIMEDIS model and the fact that the simulated results of the case study are based on empirical data of a specific environment mean that special caution must be taken in generalizing conclusions. However, the model design that includes only operational and medical processes which would be required to manage MCI casualties, can more than likely apply to other types of MCI or other DMRS, as long as victim-specific and area-specific data will be used. More experimental simulation studies must be performed to validate the SIMEDIS model.

## Conclusion

SIMEDIS represents a generic simulation model that can adequately capture the casualty flow in a DMRS and the processes related to the management of casualties in case of an MCI. It represents a tool capable of assisting DMR planners to better prepare their jurisdiction for an MCI. Using a case study of an airplane crash at an international airport, this body of research reflects the potential of SIMEDIS to model complex systems, to test different aspects of DMR, and to be used as a tool in experimental research that might make a substantial contribution to provide the evidence base for the effectiveness and efficiency of disaster medical management.

## Electronic supplementary material

ESM 1 (PDF 163 kb)

ESM 2 (PDF 416 kb)

ESM 3 (PDF 170 kb)
