# Supplementary material for: SIMEDIS: a Discrete-Event Simulation Model for Testing Responses to Mass Casualty Incidents
Source: J Med Syst. 2016 Oct 18;40(12):273. doi: 10.1007/s10916-016-0633-z (PMC5069323; doi:10.1007/s10916-016-0633-z)
Supplement: Supplementary file 1 — (PDF 163 kb) [file 10916_2016_633_MOESM1_ESM.pdf]

Appendix 1

Acronyms

ALS: advanced life support

ANOVA: analysis of variance

BLS: basic life support

CC: clinical condition

CCP: casualty collection point

DES: discrete-event simulation

DMR: disaster medical response

DMRS: disaster medical response system

ED: emergency department

EMS: emergency medical system

EMT: emergency medical technician

FMP: forward medical post

HCF: healthcare facility

MCI: mass casualty incident

MIP: medical intervention plan

MMT: mobile medical team

NATO: North Atlantic Treaty Organization

NUCA: non-urgent care area

NUCF: non-urgent care facility

PIT: paramedic intervention team

RCT: randomized controlled trial

RIT: rapid intervention team

RPM: respiratory rate, pulse rate and best motor response

SAR: search and rescue

SIMEDIS: Simulation for the assessment and optimization of medical disaster management

SME: subject matter expert

STM: Sacco triage method

S&P: stay and play

S&R: scoop and run

T1: immediate treatment triage category

T2: delayed treatment triage category

T3: minimal treatment triage category

T4: expectant treatment triage category
