# Supplementary material for: SIMEDIS: a Discrete-Event Simulation Model for Testing Responses to Mass Casualty Incidents
Source: J Med Syst. 2016 Oct 18;40(12):273. doi: 10.1007/s10916-016-0633-z (PMC5069323; doi:10.1007/s10916-016-0633-z)
Supplement: Supplementary file 2 — (PDF 416 kb) [file 10916_2016_633_MOESM2_ESM.pdf]

Appendix 2

Model translation into a computer simulation model

The conceptual model described above was implemented into a computer simulation model using a discrete-event simulation software called Arena® (version 12, Rockwell Automation). The various models are linked to Excel® files through which we had full control of all experimental input variables. Figure 1 shows a screenshot of the top-level model in Arena. Each of the boxes in the main window on the right contains a submodel (these contain the actual Arena modules). The model hierarchy is displayed on the lower left side of the screen. Figure 2 shows the Victim Pathway submodel. The coloured boxes represent standard Arena modules, while the white boxes are VBA modules. These trigger the execution of specific VBA code whenever an entity enters the module, and they are used extensively throughout the model. Figure 3 shows an example of VBA code contained within one of these modules.

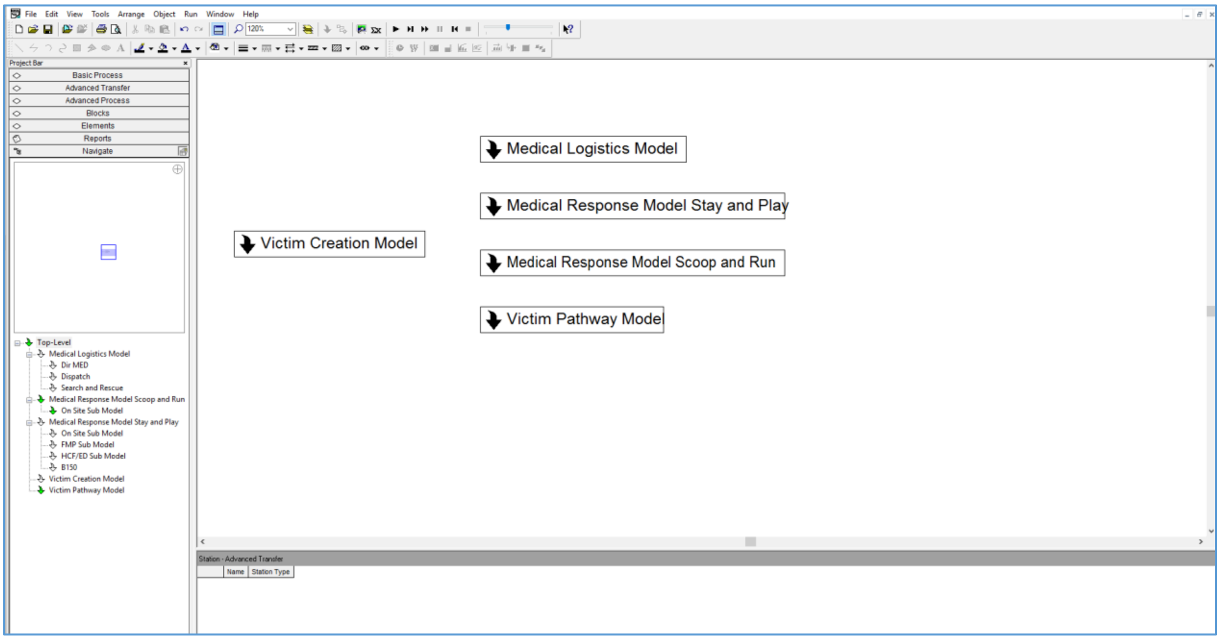

Fig. 1 The top-level model in Arena.

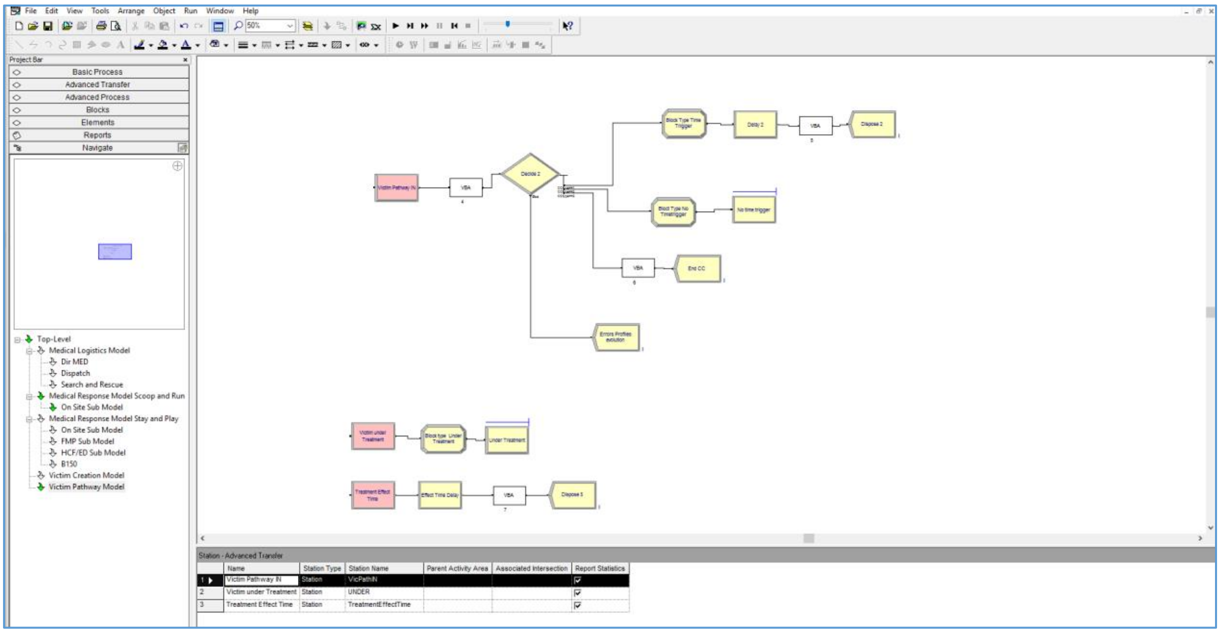

Fig. 2 The Victim Pathway sub-model in Arena.

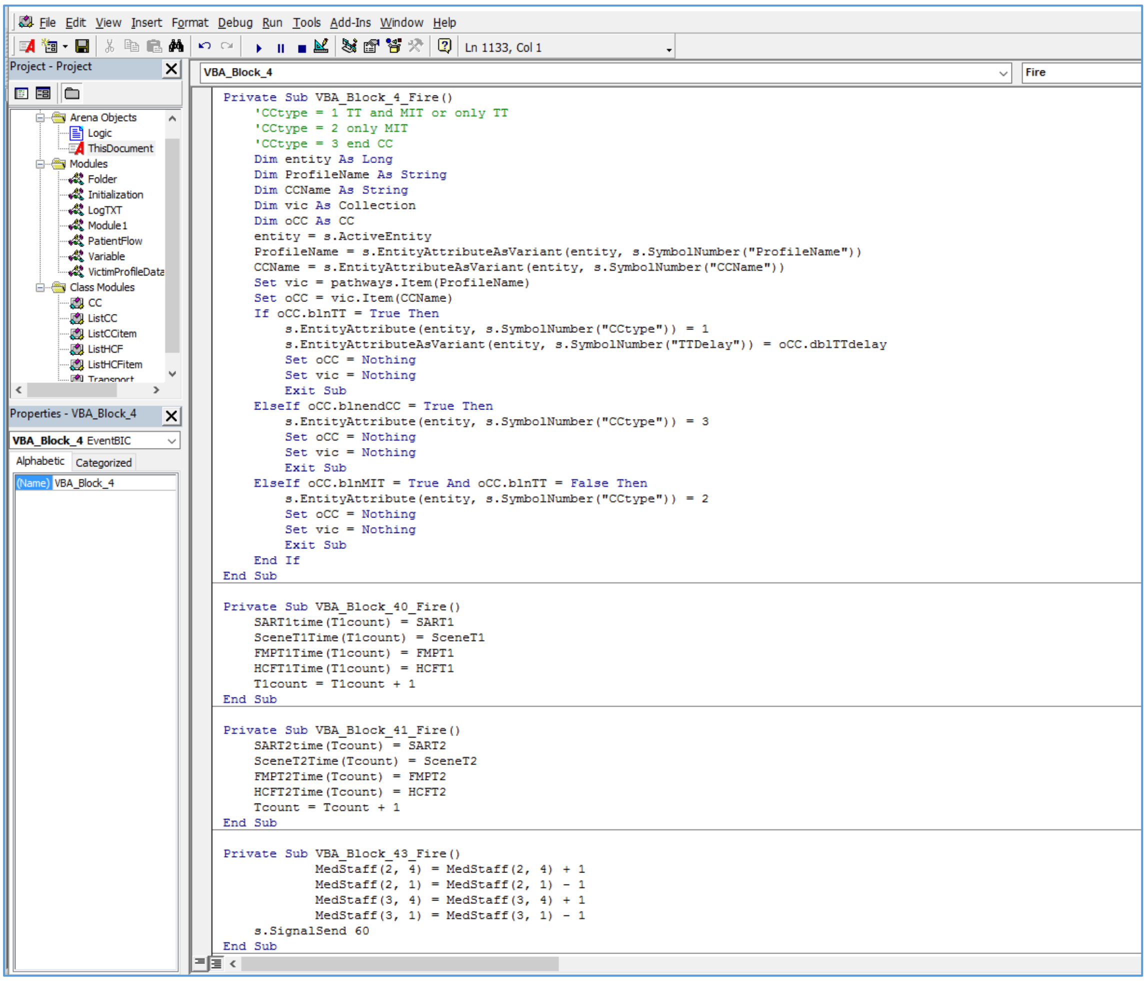

Fig. 3 Example of VBA code contained within the Arena model.

Victim creation model

The victim duplicate module creates a copy of the victim entity. Subsequently, both copies are assigned an attribute "Entity ID" which contains the identifier of the corresponding duplicate. This attribute is crucial for the interaction between the medical response model and the victim monitoring model.

Victim monitoring model

We implemented the victim monitoring model in such a way that it can handle all possible pathways with the same logic module. The duplicated victim entity enters the "Wait for Trigger " module of the victim monitoring model through a "Victim Pathway IN" station and passes the clinical condition logic test. Each CC is represented by a VBA class module and can evolve by transitions triggered by time intervals or medical interventions (Figure 4).

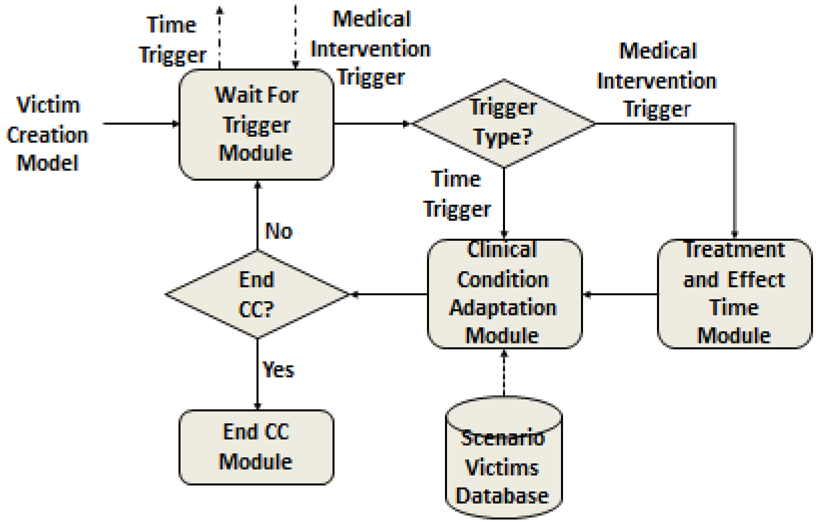

Fig. 4 Clinical condition logic.

If the CC has a time trigger, the victim entity will enter a "Time Trigger" station and goes to a delay module where it will be held until the time trigger for this CC has elapsed. The value of the time trigger delay is stored as an attribute of the victim entity. If the CC has no time trigger, the victim entity is sent to the "Medical Intervention Trigger" station and will pass the "Medical or Treatment Process" and "Effect Time" logic (see medical response model below) in the "Treatment and Effect Time" module. When the time trigger delay time or both the treatment delivery and treatment effect time has elapsed, the victim entity enters the clinical condition adaptation module which will update the parameters to those corresponding

to the new CC and will evaluate if the new CC is an end CC or not. If it is not an end CC, the victim entity returns to the “Wait for Trigger” module. Otherwise, the victim entity enters the “End CC” module and is sent to a log file. It is, however, possible that a medical treatment is initiated in the medical response model through the “Medical Process” logic before the time trigger delay has elapsed. The victim entity is then sent to the “Treatment and Effect Time” module and subsequently to the clinical condition adaptation module.

Medical Response Model

Depending on the policies and procedures applied by the disaster medical responders in the scenario, the victims will trigger a number of processes at each of the service points or during the evacuation to the HCFs. These processes are modelled for each service point based on pre-defined logic rules.

Triage and Disposition Process Logic

At each service point in the medical assistance chain the priority for treatment or evacuation of a victim is defined by the triage category of the victim, the severity of the injuries of the victim, the associated survival probability and the deterioration rate or change of survival probability over time and the arrival time in the waiting queue. Figures 5, 6 and 7 show the triage and disposition process logic in respectively the CCP, FMP and NUCA.

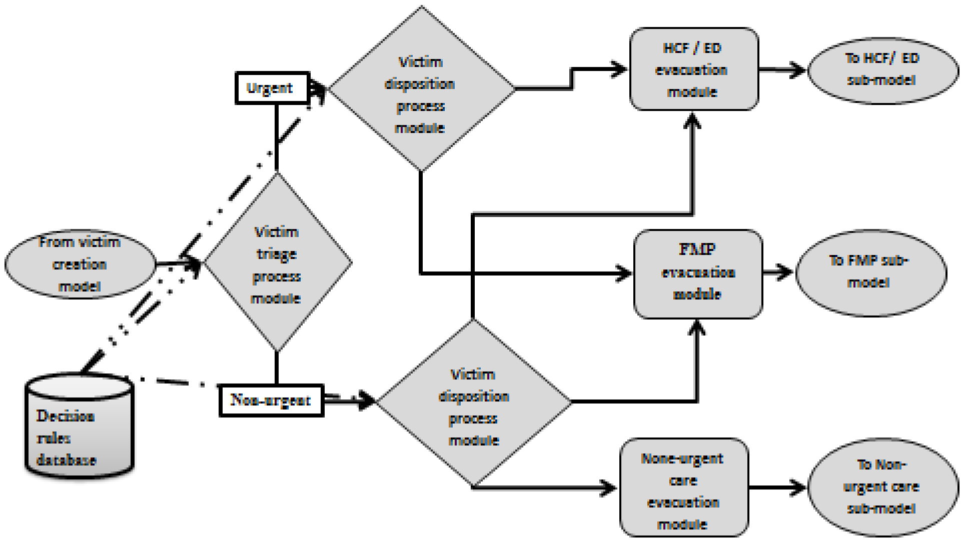

Fig. 5 Triage and disposition logic in CCP.

Victims arriving in the CCP are triaged in the urgent or non-urgent category. According to the operational policy, the victims will either be evacuated directly to HCFs and NUCFs in the "scoop and run" mode, or the urgent patients will be transferred to the FMP and the non-urgent victims to the NUCA in the "stay and play" mode.

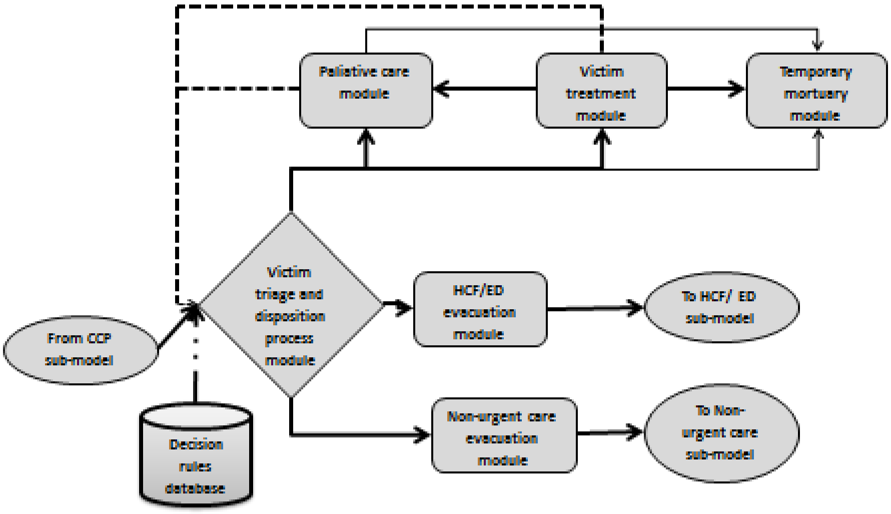

Fig. 6 Triage and disposition logic in FMP.

Urgent victims arriving in the FMP are triaged in the T1, T2 or T4 category and transferred to the appropriate treatment area within the FMP. Dead-on-arrival (DOA) victims are transferred to the temporary morgue. Surviving victims are stabilized and subsequently transported to the appropriate hospitals and specialist centres.

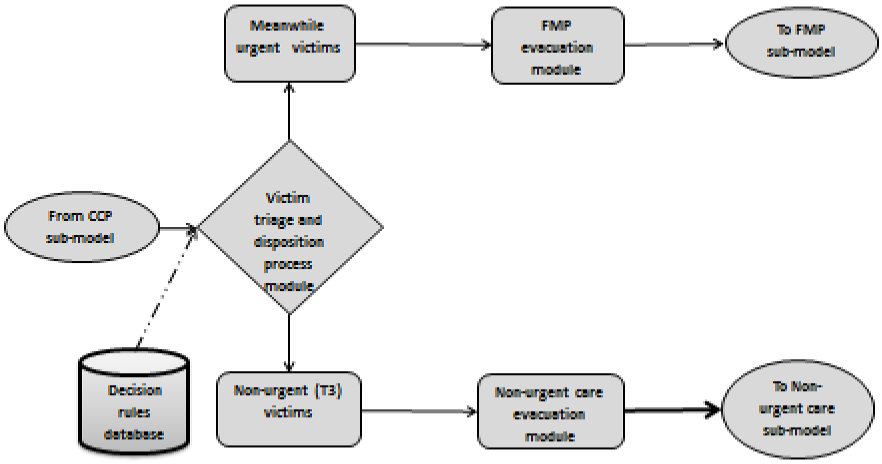

Fig. 7 Triage and disposition logic in NUCA.

Non-urgent victims arriving in the NUCA are triaged in patients whose health state deteriorated and must be transferred to the FMP, patients who can be treated in the NUCA and sent home or patients who need definitive treatment in a NUCF.

Medical or treatment process logic

The medical process logic is depicted in Figure 8.

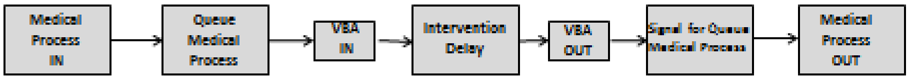

Fig. 8 Medical process logic.

The victims enter the medical process through a “Medical Process IN” station and subsequently the medical process queue. Queuing discipline is based on a priority attribute, whose exact value has been set depending on the clinical parameters of the victim and the applied triage logic. Subsequently a signal is sent which releases all the victim entities in the medical process queue. The victims, in order of priority, now enter the “VBA IN” module where both the presence of the required assets in the service point as well as their current availability is checked. If the required assets are not present or are currently being used for the treatment of other victims, the victim entity is sent back to the medical process. If the required resources are available, the victim entity is sent to the “Intervention Delay” module until the treatment delivery time has elapsed and subsequently sent to the “VBA OUT”. Finally, when the assets have been released and before the victim identity leaves the medical process logic through the “Medical Process OUT” station, a signal is sent to the medical process queue in order to release any victims awaiting treatment. Ultimately, after the updating of the CC in the victim monitoring model, the victim identity is sent back to the triage and disposition process module.

Evacuation process logic

Evacuation processes are composed of three different evacuation procedures: evacuation from the CCP to HCFs and NUCFs, from the CCP to FMP or NUCA, and from FMP or NUCA to HCFs or NUCFs.
The evacuation process logic is depicted in Figure 9.

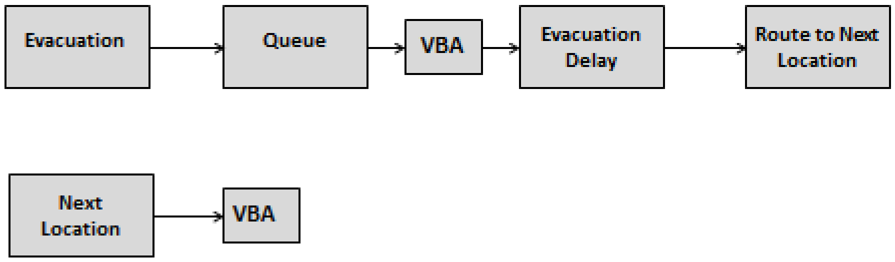

Fig. 9 Evacuation process logic.

The victims enter the evacuation process through the “Evacuation Entry” station and subsequently the evacuation queue. Queuing discipline is based on the same priority attribute as in the medical process logic. The victims, in order of priority, now enter the VBA module where the next service point for the victim and the distance and travel time are determined. The distance from CCP, FMP or NUCA to the HCFs and NUCFs is calculated by using the googlemaps APIs. The travel time between two location points are calculated by using the distance estimates and the mean ambulance speed in urban areas, on regional roads and highways. The victim entity is then delayed for the travel time before entering a route module to this service point.
Upon arrival at the service point, victim entities enter the station “Next Location” and fire the VBA code of the VBA module which creates an evacuation entity and sends this entity to the station “Evacuation Resource Travel Back”.

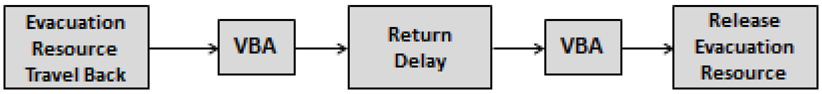

Fig. 10 Evacuation resource travel back logic.

The first VBA module in Figure 10 defines the destination of the evacuation resource and evaluates the travel time. The second VBA module releases the evacuation resources.

Since the DMR model in this study is limited to the pre-hospital phase of the response, the victims exit the simulation when they arrive at the definitive HCFs and NUCFs or no longer require care (ie dead or definitive care at the scene).
