# Supplementary material for: SIMEDIS: a Discrete-Event Simulation Model for Testing Responses to Mass Casualty Incidents
Source: J Med Syst. 2016 Oct 18;40(12):273. doi: 10.1007/s10916-016-0633-z (PMC5069323; doi:10.1007/s10916-016-0633-z)
Supplement: Supplementary file 3 — (PDF 170 kb) [file 10916_2016_633_MOESM3_ESM.pdf]

Appendix 3

Numerical (deterministic) input data of the experimental design

Table 1 Search and rescue levels

SAR level	Number of non-ambulatory victims (from 5 to 26 min)	Number of non-ambulatory victims (after 26 min)
Low	2 every 3 min	3 every 3 min
Medium	4 every 3 min	6 every 3 min
high	6 every 3 min	9 every 3 min

Table 2 Quality and quantity of pre-hospital resources

	**First wave (3-15 min)**		**Second wave (16-22 min)**	
**Level**	MMT	Ambulances	MMT	Ambulances
Normal	3	22	7	16
Low	3	16	7	22
Medium	7	16	3	22
High	7	22	3	16

Table 3 Operational time intervals

Time interval	Definition	Determination	Value (min)
Dispatch	Interval between the arrival of a call and the assignment of an EMS vehicle.	Empirical	3
Response	Interval between the departure from the EMS station and the arrival at the incident site.	Calculated	D^a x S^b / 60
Triage urgent patients	Triage of T1, T2 and T3 patients.	Empirical	0.5
Triage non-urgent patients	Triage of T3 patients.	Empirical	0.08
Loading CCP	Interval to load a patient into an ambulance at CCP.	Empirical	2
Loading FMP	Interval to load a patient into an ambulance at FMP.	Empirical	3
Unloading	Interval to unload a patient from an ambulance.	Empirical	2
Transportation	Interval between the end of loading of a patient in an ambulance at the CCP or FMP and the arrival in a HCF.	Calculated	D^a x S^b / 60
In-hospital (drop-off)	Interval between the arrival and departure of an ambulance at a HCF.	Empirical	14

(a): D is the shortest path between two location points and is calculated by using the googlemaps APIs. (b): S is the average speed of an ambulance or a MMT vehicle and is in an urban area respectively 20 km/hr and 35 km/hr, on a regional road respectively 45 km/hr and 70 km/hr and on a highway respectively 90 km/hr and 100 km/hr.

Table 4 Level of medical supervision during transport

	Low	Medium	Normal	High
TI	EMT	EP or PIT	EP or PIT	EP and PIT
T2	EMT	EMT	PIT	PIT
T3	EMT	EMT	EMT	EMT

EMT: Emergency Medical technician. PIT: emergency nurse. EP: Emergency Physician

Table 5 HCF treatment capacity at any given time

HCF	Distance	Number of adult victims/hour			Number of paediatric victims/hour		
	km	T1-T4	T2	T3	T1-T4	T2	T3
1	6.11	1	2	4	0	1	4
2	8.47	1	2	4	0	0	0
3	11.32	2	6	16	0	1	4
4	11.37	4	12	34	2	3	5
5	14.87	0	0	0	2	3	10
6	15.28	1	4	5	0	0	0
7	15.65	4	4	12	2	3	5
8	17.92	2	8	12	0	1	3
9	18.49	4	4	12	1	2	5
10	19.53	2	4	9	0	1	4
11	20.10	2	4	9	0	0	0
12	20.38	1	2	5	0	0	3
13	23.06	2	4	9	0	0	0
14	23.92	6	17	34	2	3	5
15	24.00	1	2	5	0	0	3
16	24.41	0	2	4	0	0	3
17	26.01	1	3	5	0	0	3
18	26.89	3	9	23	2	3	5
19	33.12	1	3	5	0	1	4
20	37.52	2	3	4	0	2	4

Table 6. HCF treatment capacity as indicated in its disaster plan

HCF	Number of adult victims/hour									Number of paediatric victims/hour								
	Low			Medium			High			Low			Medium			High		
	T1	T2	T3	T1	T2	T3	T1	T2	T3	T1	T2	T3	T1	T2	T3	T1	T2	T3
1	1	1	3	1	2	5	2	3	6	0	0	2	0	0	3	0	1	4
2	1	2	5	2	4	9	3	5	13	0	1	2	0	1	4	0	2	6
3	1	2	5	2	4	9	3	5	13	0	0	0	0	0	0	0	0	0
4	1	4	6	2	8	12	3	10	18	0	1	1	0	1	3	0	2	5
5	1	2	5	2	4	9	3	5	15	0	0	0	0	0	0	0	0	0
6	0	1	2	1	2	4	2	4	8	0	0	0	0	0	0	0	0	0
7	1	2	3	1	4	5	2	6	10	0	0	0	0	0	0	0	0	0
8	2	5	12	3	9	23	5	13	34	1	2	3	2	3	5	3	4	8
9	2	2	6	4	4	12	6	6	18	0	1	3	1	2	5	1	3	8
10	0	0	0	0	0	0	0	0	0	1	2	5	2	3	10	3	5	15
11	0	1	3	1	2	5	2	4	8	0	0	2	0	0	3	0	1	5
12	1	3	8	2	6	16	3	9	24	0	1	2	0	1	4	0	2	6
13	2	2	6	4	4	12	6	6	18	1	2	3	2	3	5	3	4	7
14	2	6	17	4	12	34	6	18	50	1	2	3	2	3	5	3	4	7
15	0	2	3	1	3	5	2	4	8	0	0	2	0	0	3	0	1	4
16	3	8	17	6	17	34	9	25	50	1	2	3	2	3	5	3	4	7
17	0	2	3	1	3	5	2	4	8	0	1	2	0	1	4	1	4	7
18	1	2	2	2	3	4	3	4	6	0	1	2	0	2	4	0	4	7
19	0	1	2	1	2	4	2	3	6	0	0	3	0	1	4	0	2	6
20	0	1	2	1	2	4	1	3	6	0	0	2	0	0	3	0	1	4
